# What it takes to be at the top: The interrelationship between chronic social stress and social dominance

**DOI:** 10.1002/brb3.1896

**Published:** 2020-10-17

**Authors:** Merima Šabanović, He Liu, Vongai Mlambo, Hala Aqel, Dipesh Chaudhury

**Affiliations:** ^1^ The Division of Science New York University Abu Dhabi Abu Dhabi United Arab Emirates; ^2^ Present address: Department of Experimental Psychology University of Oxford Oxford UK; ^3^ Present address: He Liu, Jiangsu Province Key Laboratory of Anesthesiology & Jiangsu Province Key Laboratory of Anesthesia and Analgesia Application Technology The Xuzhou Medical University Xuzhou China; ^4^ Present address: Department of Anesthesiology The Affiliated Hospital of Xuzhou Medical University Xuzhou China

**Keywords:** anxiety, chronic social stress, sociability, social dominance, social hierarchy

## Abstract

**Introduction:**

Dominance hierarchies of social animal groups are very sensitive to stress. Stress experienced prior to social interactions between conspecifics may be a determinant of their future social dynamics. Additionally, long‐term occupancy of a specific hierarchical rank can have psychophysiological effects which increase vulnerability to future stressors.

**Methods:**

We aimed to delineate differential effects of stress acting before or after hierarchy formation. We studied whether exposure to the chronic social defeat stress (CSDS) paradigm before a two‐week‐long hierarchy formation affected the attainment of a dominant status using the social confrontation tube test (TT). These animals were singly housed for at least one week before CSDS to decrease confounding effects of prior hierarchy experience. Additionally, we investigated whether social rank predicted vulnerability to CSDS, measured by a social interaction test.

**Results:**

In TT, mice termed as dominant (high rank) win the majority of social confrontations, while the subordinates (low rank) lose more often. Within newly established hierarchies of stress‐naïve mice, the subordinate, but not dominant, mice exhibited significantly greater avoidance of novel social targets. However, following exposure to CSDS, both lowest‐ and highest‐ranked mice exhibited susceptibility to stress as measured by decreased interactions with a novel social target. In contrast, after CSDS, both stress‐susceptible (socially avoidant) and stress‐resilient (social) mice were able to attain dominant ranks in newly established hierarchies.

**Conclusion:**

These results suggest that the response to CSDS did not determine social rank in new cohorts, but low‐status mice in newly established groups exhibited lower sociability to novel social targets. Interestingly, exposure of a hierarchical social group to chronic social stress led to stress susceptibility in both high‐ and low‐status mice as measured by social interaction.

## Graphical abstract

We aimed to delineate differential effects of stress acting before or after hierarchy formation. In newly established social hierarchies of stress‐naïve mice, the subordinate, mice exhibited significantly greater avoidance of novel social targets. Following exposure to chronic social stress, both lowest and highest ranking mice exhibit the susceptible phenotype. However, the largest change in social interaction before and after social stress was observed in the dominant mice.

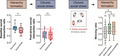

## 1 INTRODUCTION

Formation of dominance hierarchies is recognized as a universal and fundamental organizing mechanism for social animal groups (Wilson, 2000). Where resources are limited, social hierarchies determine an individual's access to food, territory, or mating partners and are readily formed due to their adaptive power of minimizing fighting among conspecifics living in close proximity (Drews, 1993). Hierarchical rank has extensive effects on physical and mental health (Bartolomucci et al., 2001; Sapolsky, 2005; Wilkinson, 1999) and therefore could become maladaptive due to the risk factors associated with living in a particular rank. Previous research has shown extensive effects of rank on behavior, including reproductive success (D’Amato, 1988), anxiety (Horii et al., 2017; but see Varholick et al., 2018), social motivation (Kunkel & Wang, 2018) and social contact (Blanchard et al., 1993), as well as on gene expression (Horii et al., 2017) and receptor expression (Lee et al., 2019).

Laboratory rodents are a particularly pertinent model organism for investigating hierarchy formation as they are very social animals and allow for studies of neuronal mechanisms underlying behavior. Both in the wild and in the laboratory, dominance hierarchies are readily observable due to the distinct patterns of behavioral characteristics in the different social ranks (reviewed in Wang et al., 2014). One of the standard tests of dominance in mice is the competitive exclusion task or the “tube test” (Fan et al., 2019), first developed to study dominance differences between inbred strains (Lindzey et al., 1961). This dyadic test offers clear and binary scoring of dominance, based on the use of space resources, that would otherwise be difficult to assess directly in the home cage.

During the formation of social hierarchies, ranks are not determined solely by intrinsic attributes, such as body size and weight, but are also affected by the environment and the prior experiences of animals. Chronic pain (Tansley et al., 2019), stress (Dixon, 1998; Krishnan et al., 2007; Park et al., 2018) and sleep history (Karamihalev et al., 2019) can have marked effects on social behaviors and dominance hierarchies. Evidence suggests that stress has a complex link to social hierarchies as it could contribute to hierarchy formation as well as arise because of hierarchy maintenance (Blanchard et al., 1995; Cordero & Sandi, 2007; Haller et al., 1999; van der Kooij & Sandi, 2012; Timmer & Sandi, 2010). Stress‐induced glucocorticoid release can increase animal's aggressiveness in acute social challenges but not in established hierarchies where intra‐colony aggression and levels of challenge are reduced (Mikics et al., 2007). This suggests that the initial establishment of a hierarchy might be particularly sensitive to modulation by glucocorticoids. Glucocorticoids have also been linked to the maintenance of memories of a defeat, but their role in the establishment of long‐term social hierarchies is more complex, depending on whether it was administered before or after the social encounter, and whether the recipient was eventually dominant or subordinate (Timmer & Sandi, 2010). Chronic elevation of glucocorticoids generally inhibits aggression (Summers et al., 2005), and repeatedly defeated males show increased basal glucocorticoid levels (Haller et al., 1999). Furthermore, intracerebroventricular glucocorticoid injection to emerging subordinate rats facilitates long‐lasting subordinate behavior (Weger et al., 2018). In general, the effects of glucocorticoids on social dominance are thought to occur via indirect effects on aggression mediated by modulatory effects on neural excitability (Joëls & de Kloet, 1992).

Extensive effects of stress are found on a behavioral, physiological, and cellular level as evidenced by alterations in synaptic plasticity (Howland & Wang, 2008), learning and memory (reviewed in Conrad, 2010), hormonal responses (Herman & Cullinan, 1997) and sleep patterns (Pawlyk et al., 2008). Nevertheless, the effects and causes of stress in relation to social hierarchies remain poorly understood. For example, it is known that prior acute stress exposure renders an animal more likely to be in a long‐term subordinate status after a conflict encounter (Cordero & Sandi, 2007). Similarly, chronic restraint stress was linked to a decreased display of social dominance in the tube test (TT; Park et al., 2018). Thus, considering that the social defeat stress (CSDS) paradigms based on the resident‐intruder aggression increase defensive and submissive behaviors in the test (intruder) animals (Martinez et al., 1998), we hypothesized that animals susceptible to CSDS would be more prone to subordinance in subsequent hierarchy formation. In addition to the effects of prior stress exposure on hierarchy formation, social rank may also affect susceptibility to future stressors. For example, a recent paper had shown that dominant mice were more susceptible to developing depression‐like behaviors following CSDS (Larrieu et al., 2017; see Larrieu & Sandi, 2018 for review). However, a depressive‐like phenotype can also be induced by long‐term subordination alone (Blanchard et al., 1995). Thus, we also wanted to elucidate whether hierarchy formation is stressful and/or capable of affecting vulnerability to further stress.

We aimed to investigate social hierarchy formation in male C57BL/6 mouse before and after exposure to stress. We tested whether chronic social stress differentially affected attainment of a dominant status in mice that are either resilient or susceptible to CSDS. We then tested whether establishing and maintaining a particular social rank prior to any further stress exposure could be stress‐inducing, as well as whether a social rank can be related to higher susceptibility or resilience to CSDS. Furthermore, we also measured the anxiety levels, presence of anhedonia, and the pattern of diurnal locomotor rhythms, as additional variables which could be influencing social behavior and stress vulnerability.

## 2 METHODS

### 2.1 Ethics

All animals were kept in Institutional Biosafety Committee (IBC) approved housing at the New York University Abu Dhabi (NYUAD) animal facility. All experimenters completed the Collaborative Training Initiative (CITI) Animal Care and Use Course, which meets United States Department of Agriculture (USDA) and Office of Laboratory Animal Welfare (OLAW) criteria for training in the humane care and use of animals in research. All animal protocols were in accordance with the National Institute of Health Guide for Care and Use of Laboratory Animals (IACUC Protocol: 150005A2) and have been approved by the NYUAD Animal Care and Use Committee.

### 2.2 Animal rearing and behavioral testing

All experiments were performed on male C57BL/6J mice (Jackson Laboratory). Upon arrival, mice were ear‐marked and allowed to acclimatize for 1 week before the onset of experiments. Animals were weighed weekly to ensure healthy weight and weight‐matching within cage groups. Behavioral experiments commenced when animals were 7 weeks old and were completed by 15 weeks of age. Retired male CD1 breeders (Charles River Laboratory) were used as resident aggressors during the CSDS paradigm. All mice were maintained under standard housing conditions at a humidity of 50 ± 10%, temperature of 23 ± 2°C and a 12 hr light/dark cycle (7 a.m.–7 p.m.), with ad libitum access to food and water. Wood shavings were used as enrichment in the home cage (green line IVCs, size: 391 × 199 × 160 mm) with social housing, unless isolation was required by the experimental protocol. Only the mice tested for the effect of stress on subsequent hierarchy formation were single housed upon arrival to avoid confounding effects of prior hierarchical rank. Within the experiment, variance was reduced by using all male mice that were age‐ and weight‐matched per cage. The same female experimenter handled mice prior to and during testing whenever possible to reduce stress and anxiety responses.

All behavioral tests were conducted during the light period of higher activity (2–7 p.m.), and the mice were habituated to the recording room and lighting conditions for at least 30 min prior to testing. Between animals, the behavioral apparatus was cleaned with MB‐10 solution (active ingredients: 20.8% sodium chlorite and 7.0% sodium dichloroisocyanurate dehydrate) for disinfection and elimination of olfactory cues.

All mice used to investigate the effects of rank on stress susceptibility underwent virus injection surgeries for neural projection tracing (data not shown). These surgeries were performed at 6‐week‐old mice who were allowed 1 week of recovery before re‐housing into novel weight‐matched groups of four. All animals recovered well from anesthesia and the surgery were healthy and displayed normal behavior. We therefore believe the surgery did not confound the experimental results reported here.

### 2.3 Social dominance tests

A minimum of 2 weeks of cohabitation were allowed for the formation of stable hierarchies, as defined by earlier studies (Varholick et al., 2018). The validity of the TT has been previously critiqued based on whether it is a true measure of dominance considering the possible confounding effects of sensorimotor capacity, learning ability, and spatial context (Bernstein, 1981; Miczek & Barry, 1975). Therefore, we have used a battery of additional dominance tests that displayed high consistency of ranking results with those obtained by the TT, thus validating that dominance was indeed the underlying variable measured in the context of our study. The rank was established first in the TT and then followed by three supplementary dominance tests as described previously (Wang et al., 2011). The supplementary dominance tests were scored blinded to the result of the TT ranking. We have only used male animals in this study as female mice are not commonly used in assessments of social dominance based on territoriality and vocalizations since females rely more on intrinsic attributes and social feedback to establish a hierarchy rather than prior social experience (Van Den Berg et al., 2015). However, some studies were able to show stable linear hierarchies in female mice, although the effect of estrus stages has to be taken into account (Rienecker et al., 2020).

#### 2.3.1 Social confrontation TT

Our customized automated TT system (Clever Sys Inc.) consisted of a clear plexiglass tube 55 cm in length and 2.5 cm in diameter, sufficiently wide for one mouse to walk through but not for two mice to pass each other. The tube was connected to a 10 × 10 cm box on each side. The box in which a mouse was initially placed was called the “starting box,” for which the box at the other end would be the “goal box.” Automated doors were placed at the box exits and in the middle of the tube. All animals underwent a two‐day training phase to learn how to enter the tube, be within 3 cm of the middle door to open it and initiate a trial, and then to pass to the goal box on the other side. On each of the 2 days, animals had to complete a total of 10 trials, five starting on each side of the tube, making a total of 20 trials for the whole training phase. If the mouse remained stationary for longer than 5 s, or began retreating, a gentle push from behind was used to direct movement toward the middle door. The use of food deprivation and food reward previously showed no effect on animals’ motivation to complete the task (Fan et al., 2019) and were therefore not used in this study.

**FIGURE 1:**
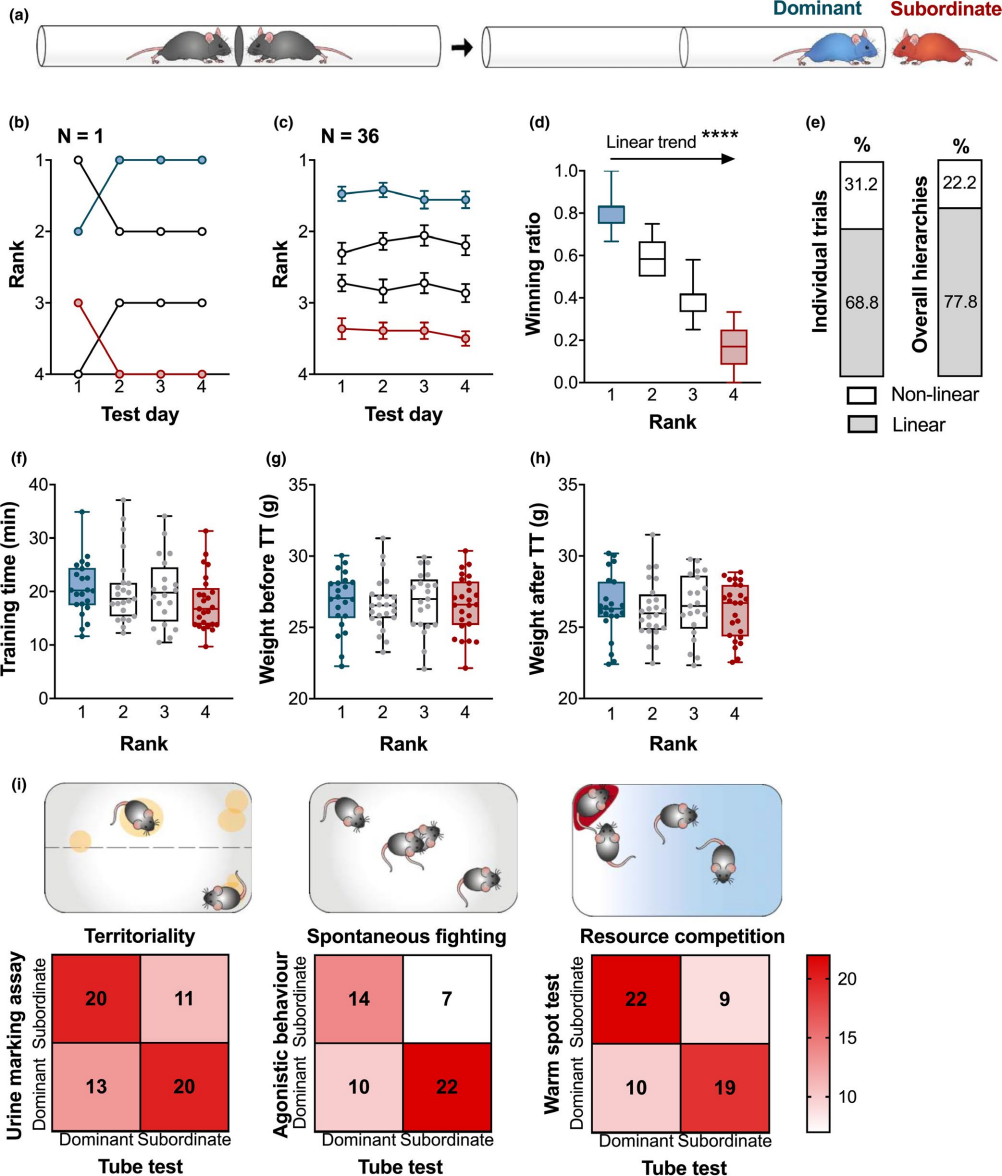
Social confrontation tube test was validated to yield predominantly linear and stable hierarchies consistent with other measures of dominance. (a) Diagram of a confrontation trial. The subordinate mouse retreated from the tube first. (b) Representative image of the rank stability in a cage of four mice over the 4 days of testing. (c) Overall rank stability shows the average rank of animals belonging to each rank group (as determined at the end of TT) calculated for each day of testing for a total of *N* = 25 cages. (d) Winning ratio of ranked mice (*N* = 34–37 per rank) showed a significant linear trend from the most dominant to the most subordinate. (e) Individual daily trials and final hierarchies were predominantly linear. (f) The total time spent in the tube during a two‐day training phase did not differ between ranks (*N* = 20–25 per rank). (g) Weight was not a factor in establishing dominance as groups are weight‐matched prior to TT (*N* = 20–25 per rank). (h) Weights of ranked mice were comparable after testing (*N* = 20–25 per rank). (i) TT correlated with rankings from three other dominance tests: urine marking assay, UMA (*p* = .0509); agonistic behavior test, ABT (*p* = .0229); and the warm spot test, WST (*p* = .0090). Data are shown as mean ± *SEM* or box plot with whiskers denoting the min. and max. values. ****p < .0001

During the testing phase, each group of animals underwent the TT once and repeated it daily thereafter. Our pilot experiments used a seven‐day testing phase, but the animals started displaying signs of stress and reduced task motivation in the later days, so we opted for the four‐day testing instead (i.e., each group did four TTs in total in four consecutive days). On each day of the testing phase, each mouse explored the tube once from each side prior to starting the confrontation trials. Using a round‐robin design, all pairs of mice from the same cage were tested (six pairs per social group of four mice; each mouse undergoes three TTs in total on 1 day). During the trial, both mice were guided into the tube simultaneously from their respective starting boxes. The starting side for each mouse alternated between trials. When both mice were within 3 cm of the middle door, the door opened, and the social confrontation trial began. The trial ended when one of the mice retreated with all four paws to its starting box, therefore becoming the “loser” or the subordinate (Figure 1a). The mouse that forced its cage mate to retreat was termed the “winner” or the dominant. In between trials, mice were kept in separate clean holding cages. The confrontation trials were repeated for four consecutive days with a randomized order of the pairs and the cages. The experimenter remained stationary during each trial in a designated position in the room to maintain cue consistency.

The winning ratio was calculated as the number of all trials won by that mouse divided by the total number of trials. This determined the index of overall dominance where rank 1 and rank 2 mice (winning ratio >0.5) were the dominant, while rank 3 and rank 4 mice (winning ratio <0.5) were the subordinate mice.

#### 2.3.2 Territory urine marking assay

Mice are territorial animals and urinary scent marking serves to indicate territorial boundaries and dominance status, strongly influencing their aggressive interactions (Arakawa et al., 2008). The number of scent marks can predict both aggression scores and social dominance status in mice (Drickamer, 2001). Using the round‐robin design, each of the six possible pairs from the same home cage was tested (two pairs per day for the total of 3 days) following a protocol established by Wang et al. (2011). The number, size, and the distance of urine marks from the central partition were scored blinded to the tube test result. “Dominant” males were identified as those making more urine marks and/or close to the partition, whereas “subordinate males” were those urinating in fewer locations and/or further away from the partition. A total of 20 cages of animals were tested, with 16 yielding unambiguous dominant‐subordinate relationships within the group.

#### 2.3.3 Warm spot test

This test was adapted from Zhou et al. (2017). A rectangular plastic cage 29.5 × 18 cm was placed on ice, cooling the floor of the cage to 0–4°C. The mice were first habituated to the cold cage for 30 min. Then, they were transferred to a new cold cage with a 5 × 5 cm warm pad heated to 34°C. Correct temperatures were ensured by monitoring with an infrared thermometer. As the warm spot was big enough to permit the stay of only one adult mouse, the competition of the tail‐marked four mice for the warm spot was videotaped for 20 min and the time each mouse spent occupying the warm spot was analyzed. Dominance was scored by the longer time spent on the warm spot and blinded to the tube test result.

#### 2.3.4 Agonistic behavior test

Mice group‐housed together for an extended period will not exhibit extensive aggressive behaviors toward each other. Others have reported that agonistic behavior is potentiated upon placing the animals in a new cage which requires the animals to claim the new territory (Wang et al., 2011). We observed increased instances of agonistic interactions immediately after returning the animals to their home cage after behavioral testing, presumably due to the need to reinforce their status upon re‐entering their territory. Accordingly, tail‐marked mice were videotaped for 15 min upon returning to the home cage following either the urine marking assay (UMA) or the warm spot test (WST), recording the occurrence of spontaneous fighting and offensive or defensive behavior. Offensive behaviors were characterized as chasing and attacking, while the submissive behaviors included flight, freezing, and submissive posture (exposed abdomen, limp forepaws, and head angled up). In most cases, only one mouse out of four in the group would initiate an attack. Agonistic behavior was observed in 13 out of 23 cages tested and was scored blinded to TT results.

### 2.4 Inducing and characterizing chronic social stress

#### 2.4.1 Chronic social defeat stress

The paradigm was adapted from Golden et al. (2011) where the duration of stress exposure was extended to 15 days. CD1 mice were screened for aggression in their home cage and rescreened prior to starting CSDS, excluding nonaggressive mice. An experimental mouse was placed into the home cage of a CD1 aggressor mouse for 10 min during which time it endured several bouts of physical attacks by the aggressor. The CD1 and experimental mouse were then maintained in sensory contact for 24 hr using a perforated plexiglass partition dividing the resident home cage in two. On each consecutive day, the experimental mice were exposed to a new CD1 mouse home cage to avoid habituation to the aggressor. The repeated social defeats were performed between 3 and 5 p.m. Control mice were housed in pairs within a cage setup identical to that of CSDS mice, with the two mice continuously separated by the perforated Plexiglass divider. Control animals were removed from the room during the defeat sessions, to avoid exposure to stress‐induced vocalizations by their conspecifics. Twenty‐four hours after the last defeat session, the mice were taken out of the CSDS cages and single housed in new cages, allowing a minimum 3 hr of habituation before starting the social interaction test.

#### 2.4.2 Social interaction test

A two‐stage social interaction (SI) test was adapted from Golden et al. (2011). In the first 2.5 min‐long nonsocial session (no social target present), the mouse could freely explore a square‐shaped arena (42 × 42 cm) containing a clear Plexiglass cage with a wire mesh (10 × 6.5 cm) placed on one side of the arena. In the second 2.5 min‐long social session (with a neutral novel social target present), the experimental mouse was introduced back into the arena with an unfamiliar CD1 mouse contained behind a wire mesh cage. Social interaction deficits are transferrable across species, observed with an unfamiliar CD1 as well as a C57 social target (Krishnan et al., 2007). Between the nonsocial and social sessions, the mouse was removed from the arena and placed in his home or neutral cage. Video tracking software (TopScan, Clever Sys Inc., RRID:SCR_017141, http://cleversysinc.com/CleverSysInc/?csi_products=topscan-suite) was used to measure the amount of time the experimental mouse spent in the “interaction zone” (24 × 15 cm surrounding the wire mesh cage), “corner zone” (9 × 9 cm from opposite walls), as well as the total distance travelled by the mouse. The social interaction ratio (SI ratio) was obtained by dividing the time spent in the interaction zone in the social session divided by the object session. Susceptibility to stress was characterized by a reduction in SI ratio to values below 1.0, indicating social avoidance. Two separate populations were defined as “stress‐susceptible” (post‐CSDS SI ratio < 1.0) and “stress‐resilient” (post‐CSDS SI ratio > 1.0).

#### 2.4.3 Sucrose preference test

To test whether CSDS induced anhedonia, we measured the sucrose preference of a subset of animals in our studies. Animals were single housed and habituated to two bottles of 1% sucrose for 2 days, followed by a 24 hr‐period of food and water deprivation. In the 3 hr test period, the animals were given one bottle of 1% sucrose and one bottle of water, with bottle positions switched halfway through the experiment, to control for any side‐preference. The sucrose and water bottles were weighed before and after the test, recording the total consumption of each liquid. Sucrose preference was defined as total sucrose consumption divided by total liquid consumption (water and sucrose).

#### 2.4.4 Wheel‐running assay

Previous work has shown that mice exposed to stress exhibited abnormal diurnal rhythms in physiology and behavior (Bunney et al., 2015). Additionally, hierarchy establishment can affect the sleep architecture of dominant and subordinate mice (Karamihalev et al., 2019). This would suggest that stress exposure in the form of CSDS or rank maintenance could lead to disruptions in daily rhythms. Moreover, the running wheels are commonly used as a measure of circadian activity rhythms, but evidence suggests that wheel running is also rewarding to rodents (see Novak et al., 2012 for review) and can therefore potentially be used as a surrogate for motivated physical activity. Wheel‐running assay was therefore used as an additional test of rank‐ and/or CSDS‐related stress (similar to SI and sucrose preference test [SPT]) in a subset of animals.

Voluntary wheel‐running cages were placed in circadian cabinets (Phenome Technologies) with a maximum of six cages per row. The light and temperature of the chambers were controlled by the ClockLab Chamber Control software (ACT‐500, http://actimetrics.com/products/clocklab, RRID:SCR_014309). The running wheel cages are available from Actimetrics (model: ACT‐551‐MS‐SS) and consisted of a Tecniplast model 1144B cage bottom (33.2 × 15 × 13 cm) and a wire bar lid. The wheel was stainless steel, 11 cm inside diameter, 5.4 cm wide, with 1.2 mm wide bars placed 7.5 mm apart. The infrared (clickless) sensor clipped onto the lip and rail of the cage and detected the spokes of the wheel passing by. The sensor was connected via a cable to the ClockLab digital interface (ACT‐556). ClockLab Data Collection software (ACT‐500) registered each revolution of the wheel as a count. The number of counts per minute of each wheel was recorded and the final analysis was done on the total counts per hour with ClockLab Analysis Version 6 (ACT‐500). The total of 1 week of recording was used.

Some studies suggest voluntary wheel running has anti‐depressive and anti‐anxiety‐like effects, but the review of previous research shows that a minimum of 3–4 weeks of unrestricted access are required for such effects to be significant, (Novak et al., 2012). Considering this protocol includes only 1 week of wheel running, we do not expect that such behavioral alterations presented a significant confounding variable for the experiments that followed the wheel‐running activity assay.

### 2.5 Anxiety tests

All animals were handled for a minimum of 2 days prior to onset of baseline anxiety measurements, allowing the mice to habituate to the experimenter interaction. TopScan (Clever Sys Inc.) video tracking system recorded the time spent in each zone, as well as bouts of entering each zone and total distance travelled as measures of exploration.

#### 2.5.1 Open field test

The apparatus consisted of the same arena used for the SI test (42 × 42 cm), with the “centre” zone defined as the inner 32 × 32 cm. Under red light, mice were placed into the centre of the arena and allowed 15 min of free exploration. Thigmotaxis in the open field (OF) was defined as the percentage of testing time the animal spent near the walls of the arena and not in the centre.

#### 2.5.2 Elevated plus maze test

A grey polyvinyl chloride (PVC) apparatus was in the “+” configuration comprising of two open arms (34 × 6 cm) perpendicular to two closed arms (34 × 6 cm with 21.5 cm tall walls) with a centre zone (6 × 6 cm). The entire apparatus was 60 cm above the ground and illuminated by red light. The animal was placed in the centre zone, opposing the experimenter, and allowed 5 min of free exploration. Thigmotaxis in the elevated plus maze (EPM) was defined as the percentage of testing time the animal spent in the closed arms of the maze.

### 2.6 Statistical analyses

Animals were randomly assigned to treatment groups, but cage groups were matched by weight. Where possible, the experimenter was blinded to treatments. Statistical analyses were performed using GraphPad Prism software v7 (https://www.graphpad.com, RRID:SCR_002798). All values are given as a mean ± *SEM*. All statistical tests were two‐tailed and the significance was assigned at *p* < .05. The D’Agostino‐Pearson omnibus normality test and Brown–Forsythe test were used to test normality and equal variances between group samples, respectively. When normality and equal variance between sample groups was achieved, ordinary one‐way ANOVA (followed by Tukey's multiple comparisons test), repeated measures two‐way ANOVA, unpaired or one sample *t*‐tests were used. Where normality or equal variance of samples failed, Mann–Whitney *U* test, Kruskal–Wallis test with Dunn's post hoc multiple comparison, or Wilcoxon signed rank test was performed. Linear regression and Fisher's exact tests were used for correlation and contingency analyses.

### 2.7 Experiment 1: Effect of CSD on novel hierarchy formation

After a baseline SI test, one cohort of animals (*n* = 80) underwent 15 days of CSDS, followed by another SI test. A subset of animals also underwent SPT and a one‐week wheel‐running assay. Based on the SI scores (see Section 2.4.2), CSDS‐exposed animals were split into two groups, the stress‐resilient or stress‐susceptible. One mouse died during CSDS making *n* = 76 for subsequent analyses (as three stress‐naïve males due to be the cage mates were also excluded). The mice were then housed in weight‐matched groups such that there was one CSDS‐stressed mouse together with three stress‐naïve controls in one cage (total of *N* = 19 cages). After 2 weeks of hierarchy formation, TT and supporting dominance tests were performed as described in Section 2.3. Anxiety was measured prior to starting and after completing all behavioral tests.

### 2.8 Experiment 2: Effect of social status on stress susceptibility

To delineate whether newly formed hierarchies would predispose a certain rank to greater stress susceptibility, a second cohort of mice (*n* = 68) was first matched by weight and group‐housed immediately after trait anxiety tests (total of *N* = 176 cages). After ranks were determined in dominance tests and a baseline SI was recorded, all ranks underwent CSDS with a subset of mice (*n* = 12) serving as CSDS‐controls (and therefore being excluded from further comparison based only on CSDS‐exposed mice). We report death of one control mouse during CSDS due to unrelated sickness (its dominance data were still used for TT validation). This did not impact subsequent analysis of rank 1 versus rank 4 differences. The same tests of stress were performed as in Experiment 1, followed by state anxiety recordings. To minimize the variability due to different hierarchical structures, main comparison was limited to the clear alphas or omegas of a group, the rank 1 (*n* = 17) and rank 4 mice (*n* = 16) respectively.

## 3 RESULTS

### 3.1 Hierarchy formation and TT validation

Following 2 weeks of agonistic activity, establishment of social hierarchies among cage mates was determined in a battery of social dominance tests. In the TT, the measured hierarchies were consistent over the four‐day testing period, both within and across groups respectively (Figure 1b,c), and exhibited a linear trend from the most dominant to the most subordinate animal (Figure 1d: One‐way ANOVA *F*(3,140) = 361.9, *p* < .0001: post‐test for linear trend slope = −0.2134 ± −0.0065, *R* ^2^ = .8858, *F*(1,140) = 1,086, *p* < .0001). In a linear hierarchy, the top individual (“alpha”) dominates over all others. Each subsequent rank is singly occupied, down to the most subordinate mouse (“omega”) that is dominated by all other members. Nearly 78% of hierarchies observed in this experiment were linear (Figure 1e), but other hierarchical structures were observed, such as nontransitive and despotic (one alpha with other members not having clear ranks). The TT rank is not induced by the testing procedure, as the time spent in the tube during the training phase was not an indicator of success during testing (Figure 1f: Kruskal–Wallis *H*(3) = 3.83, *p* = .2804) and neither was the weight profile before (Figure 1g: One‐way ANOVA *F*(3,88) = 0.3467, *p* = .9576) or after testing (Figure 1h: One‐way ANOVA *F*(3,88) = 0.1442, *p* = .9331). The validity of TT‐obtained ranks is supported by correlation with other dominance measures that highlight different manifestations of dominance behavior. Dominance ranks from the TT were consistent with ranks obtained via three other methods: territoriality in the UMA, spontaneous fighting in the agonistic behavior test (ABT), and resource competition in the WST (Figure 1i: Fisher's exact *t*‐tests (2‐sided) UMA *p* = .0509, ABT *p* = .0229, WST *p* = .0090). The degree of correlation ensures that dominance is the common underlying factor being measured. Ranked mice belonged to two experimental groups based on exposure to CSDS before or after hierarchy formation, as described in Sections 2.7 and 2.8.

### 3.2 Experiment 1: Effect of CSD on novel hierarchy formation

**FIGURE 2:**
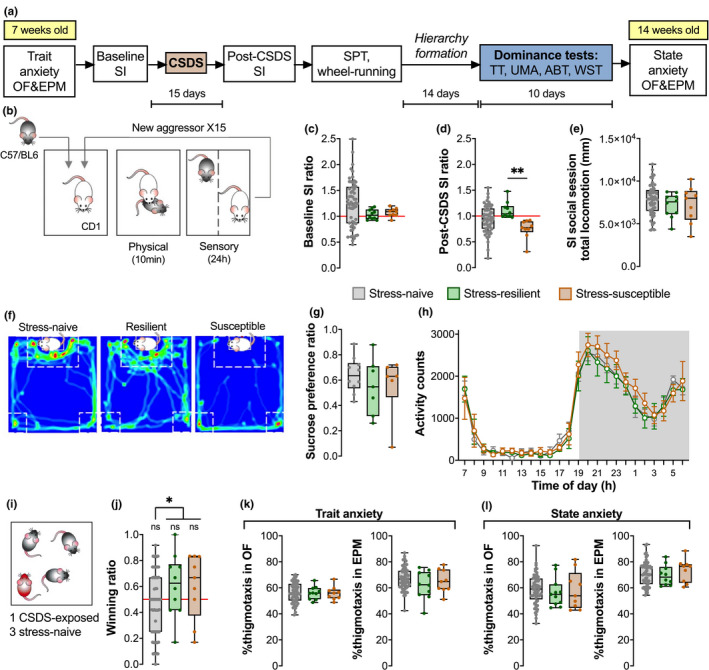
Chronic social defeat stress results in two distinct populations based on social interaction profiles that did not differ in subsequent hierarchy formation. (a) Timeline of behavioral studies investigating the effect of chronic stress on subsequent hierarchy formation. The age of mice is given in yellow boxes. (b) 15‐day CSDS paradigm consisted of daily sessions of 10 min physical stress followed by 24 hr of sensory stress. (c) Before chronic stress exposure, SI ratios did not differ between groups. (d) After CSDS, a subset of mice termed “stress‐susceptible” exhibited an SI ratio lower than that of “stress‐resilient” mice. (e) Levels of exploration, as measured by total distance travelled during the social session of the SI test, were comparable across stress groups. (f) Representative traces of the time spent interacting with a social target show that stress‐susceptible mice avoided the interaction zone around the social target mouse and escape to the corner zones. (g) None of the groups exhibited anhedonia following CSDS. (h) All groups exhibited similar daily wheel‐running activity profiles. (i) One CSDS‐exposed mouse was group‐housed with three stress‐naïve controls. (j) There was no difference in dominance between stress‐exposed groups. (k,l) Anxiety profiles in both OF and EPM anxiety tests did not differ between stress groups either before or after behavioral testing. *N*_Stress‐naïve_ = 57, *N*_Resilient_ = 10, *N*_Susceptible_ = 9 except for (g,h) where *N*_Stress‐naïve_ = 10, *N*_Resilient_ = 7, *N*_Susceptible_ = 6. Data are shown as mean ± *SEM*. **p < .01. ABT, agonistic behavior test; CSDS, chronic social defeat stress; EPM, elevated plus maze; OF, open field; SI, social interaction; SPT, sucrose preference test; TT, tube test; UMA, urine marking assay; WST, warm spot test

The full experimental timeline is shown in Figure 2a.

#### 3.2.1 CSDS induces reduced social preference in a subset of stress‐susceptible mice

Chronic social stress was induced using the CSDS procedure based on social conflict, as described in Section 2.4.1 (Figure 2b). In the baseline SI test, mice exhibited a normal distribution of social preferences (Figure 2c: One‐way ANOVA *F*(2,73) = 1.701, *p* = .1897). Following CSDS, animals could be distinguished as belonging to two separate groups – stress‐susceptible (socially avoidant) mice were recognizable by their lower SI ratios (<1.0) that were significantly different from the SI ratios of stress‐resilient (social) mice (Figure 2d: Kruskal–Wallis *H* = 11.45, *p* = .0033; Dunn's multiple comparisons naïve‐resilient *p* = .1213, naïve‐susceptible *p* = .0529, resilient‐susceptible *p* = .0022). This reduced SI ratio was not due to reduced exploration (Figure 2e: One‐way ANOVA *F*(2,73) = 0.5333, *p* = .4613) but instead reflected the tendency of stress‐susceptible mice to avoid the interaction zones of the SI arena (Figure 2f). Reduced social preference in this case may not be a marker of a depressive‐like phenotype as mice did not exhibit anhedonia in the SPT (Figure 2g: One‐way ANOVA *F*(2,20) = 0.5641, *p* = .5776) or aberrant wheel‐running activity (Figure 2h: Two‐way RM ANOVA stress group effect *F*(2,20) = 0.3953, *p* = .6786). Accordingly, “stress susceptibility” was therefore used as a measure of stress‐induced social avoidance.

#### 3.2.2 CSDS did not diminish success of stress‐exposed mice in subsequent hierarchy formation

Following CSDS, the mice were weight‐matched and group‐housed such that there is one stress‐resilient (*N* = 10) or stress‐susceptible (*N* = 9) mouse together with three stress‐naïve controls (total *N* = 57) per cage (Figure 2i). After 2 weeks of hierarchy formation, winning ratios obtained in the TT were compared between stress groups. Surprisingly, neither stress‐resilient nor stress‐susceptible mice were more likely to be subordinate and their average winning ratios were comparable (Figure 2j: stress‐naive Wilcoxon Signed Rank median = 0.5, *p* = .2239; One sample *t*‐test, resilient *t*(9) = 1.423, *p* = .1885 and susceptible *t*(8) = 1.214, *p* = .2594). Our post hoc exploratory analyses suggest that CSDS‐exposed mice occupy the more dominant positions in their respective cohorts (Figure 2j: stress‐naïve vs. CSD‐exposed Mann–Whitney *U* = 365, exact *p*‐value = .0330), but more rigorous follow‐up studies with higher power would be needed for conclusive results.

#### 3.2.3 Neither trait nor state anxiety could be used as a predictor of stress susceptibility

Open field and EPM anxiety tests were performed prior to starting behavioral manipulations to establish the characteristic of the individual (trait anxiety). Moreover, OF and EPM tests were performed after exposure to the CSDS and dominance test to determine the effects of experiencing chronic stress and hierarchy formation on anxiety (state anxiety). Both OF and EPM tests use the measure of thigmotaxis, defined as the tendency to remain close to walls or enclosed spaces, as a proxy for high anxiety. All experimental groups remained comparable to each other at both time‐points (One‐way ANOVAs, Figure 2k: OF: *F*(2,73) = 0.03089, *p* = .9696; EPM: *F*(2,73) = 1.332, *p* = .2704; Figure 2l: OF: *F*(2,73) = 0.2323, *p* = .7933; EPM: *F*(2,73) = 0.4725, *p* = .6254). Explorative behaviors were not affected since behavioral measures such as bouts of zone entries and total locomotion were consistent between groups in all tests (Figure S1).

### 3.3 Experiment 2: Effect of social status on stress susceptibility

**FIGURE 3:**
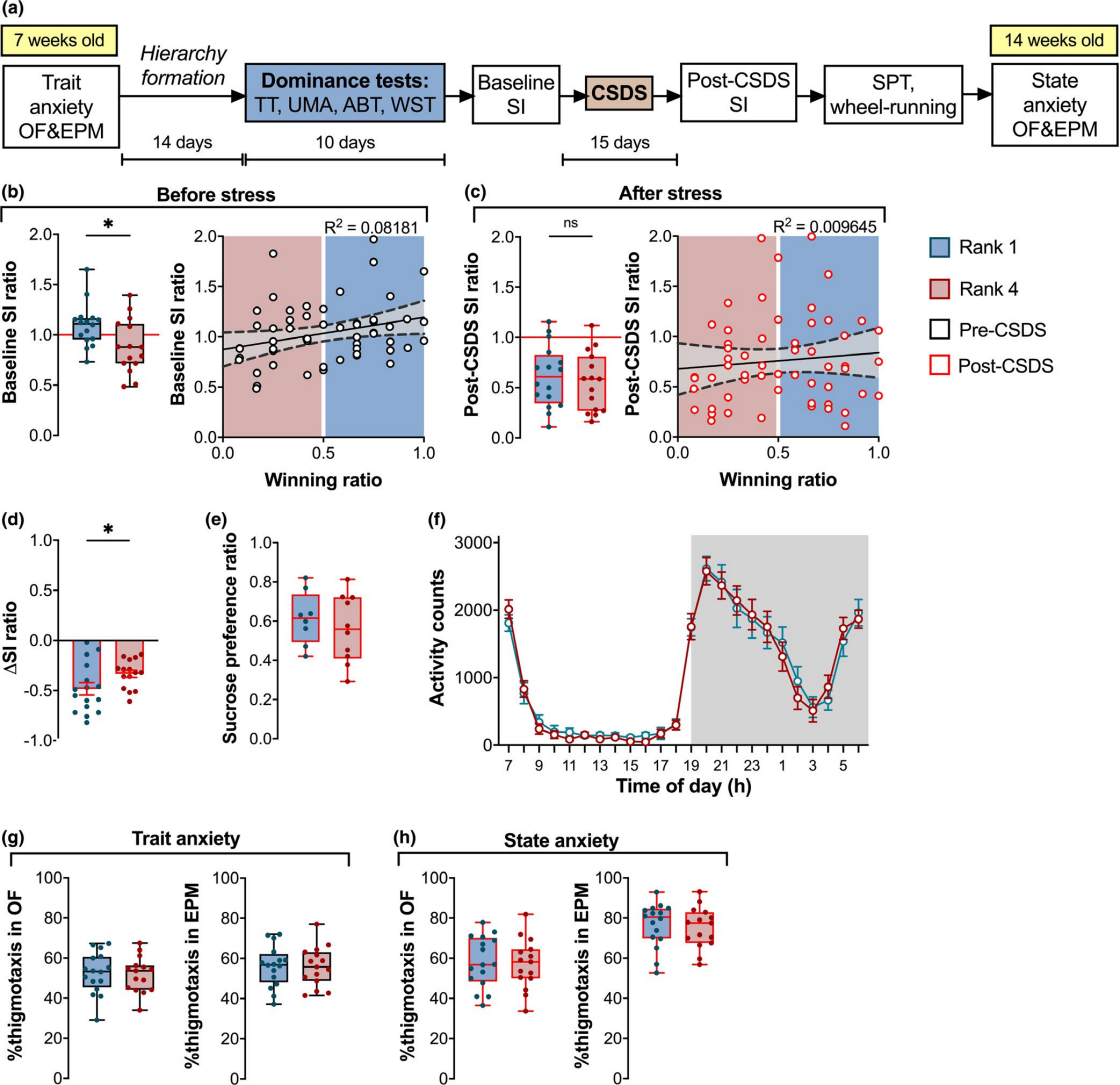
Baseline, but not post‐CSD, sociability is rank‐dependent. Change of social preference in rank 1 mice was significantly greater after CSD compared to rank 4 mice. (a) Timeline of behavioral studies investigating the effect of dominance status on susceptibility to chronic social stress. The age of mice is indicated in yellow boxes. (b) Pre‐CSDS: Following hierarchy formation, the winning and baseline SI ratios exhibited positive correlation, with dominant mice exhibiting higher sociability. (c) Post‐CSDS, winning and SI ratios of ranked mice did not differ significantly. (d) Rank 1 displayed a higher change in SI ratio following CSDS. (e) Rank 1 and rank 4 mice did not exhibit anhedonia in the SPT. (f) Daily wheel‐running activity profiles were similar across ranks. (g,h) There were no significant differences between groups in either trait or state anxiety tests. *N*_rank1_ = 16, *N*_rank4_ = 15, except for (e) where *N*_rank1_ = 8, *N*_rank4_ = 10 and (f) where *N*_rank1_ = *N*_rank4_ = 12. Data are shown as mean ± *SEM*. *p < .05. ABT, agonistic behavior test; CSDS, chronic social defeat stress; EPM, elevated plus maze; OF, open field; SI, social interaction; SPT, sucrose preference test; TT, tube test; UMA, urine marking assay; WST, warm spot test

The full experimental timeline is shown in Figure 3a.

#### 3.3.1 Social dominance could be a predictor of sociability but not of stress susceptibility

Following 2 weeks of hierarchy establishment and maintenance, rank 4 (subordinate) mice had a significantly lower SI ratio than the rank 1 (dominant) mice Figure 3b left: mean difference = 0.21 ± 0.09; Unpaired *t*‐test *t*(29) = 2.374, *p* = .0245). Overall, the TT winning ratio positively correlated with the pre‐CSDS SI ratio (Figure 3b right: Linear regression *F*(1,54) = 4.811, *p* = .0326, *R* ^2^ = .08181), with the dominant ranks exhibiting higher sociability than the subordinate ranks. In contrast, after CSDS, there was no difference between the ranks (Figure 3c left: mean difference 0.06 ± 0.11; Unpaired *t*‐test *t*(29) = 0.5417, *p* = .5922). Additionally, there was no correlation between SI and winning ratio (Figure 3c right: Linear regression *F*(1,54) = 0.5259, *p* = .4715, *R* ^2^ = .009645). While both ranks showed a reduction in the SI ratio following CSDS, the change was significantly greater for dominant mice (Figure 3d: Unpaired *t*‐test *t*(29) = 2.058, *p* = .0487). This raises the possibility that dominant mice were more severely affected by the experience of chronic stress, or that the social interaction ratio of subordinate animals, being lower already at the start, exhibited a floor effect after CSDS. There were no differences in sucrose preference (Figure 3e: Unpaired *t*‐test *t*(16) = 0.7069, *p* = .4898) or wheel‐running activity between ranks after stress exposure (Figure 3f: Two‐way RM ANOVA stress group effect *F*(1,22) = 0.008877, *p* = .9258).

#### 3.3.2 Anxiety profiles were not rank‐ or stress‐experience‐dependent

Thigmotaxis profiles in OF and EPM were not different between ranks 1 and 4 either before or after CSDS (Figure 3g: Unpaired *t*‐test OF: *t*(29) = 0.2184, *p* = .8287; EPM: *t*(29) = 0.03974, *p* = .9686; Figure 3h: Unpaired *t*‐test OF: *t*(29) = 0.2516, *p* = .8031; EPM: *t*(29) = 0.3931, *p* = .6971). Measures of explorative and locomotive behavior were also comparable in all cases (Figure S2). Hence, we cannot report any rank‐dependent differences in anxiety measures.

## 4 DISCUSSION

### 4.1 Chronic exposure to social defeat did not render mice subordinate

We anticipated that CSDS exposure may have differential effects on hierarchy formation such that susceptible mice would be more prone to subordination as they exhibit compromised ability to handle stressful conflict situations. In contrast, we predicted that resilient mice would exhibit higher dominance as they may have acquired a more adaptive strategy for adjusting to new social cohorts, which enabled them to overcome any putative adverse effects of CSDS. However, we found that both susceptible and resilient mice exhibited comparable dominance levels, as evidenced by the equal distribution of the winning ratios.

While depression is mainly associated with despondency and social withdrawal, aggression is also a common symptom in human depressive states (Van Praag, 1998). Moreover, the chronic unpredictable stress model was reported to increase aggression, hostility, and social dominance in rodents (Yang et al., 2015). Our study did not quantify push, retreat, and resistance behaviors of animals within the tube. Therefore, we cannot exclude the possibility that CSDS‐exposed mice win via a more or less effortful strategy than the stress‐naïve controls, for example by “freezing” in the tube instead of pushing until the opponent retreats. CSDS could also lead to an increase in the levels of glucocorticoids, which might play a key role in shaping the behavioral trajectories leading to social rank attainment via glucocorticoid receptor activation, specifically in the nucleus accumbens (Papilloud et al., 2020). Additional measures of animals’ agonistic propensity could delineate whether increased aggression or other behavioral strategies would account for these observations.

Glucocorticoids are reported to modulate neurobehavioral plasticity engaged in shaping social subordination (Weger et al., 2018). Thus, we predicted that chronic stress, which would lead to long‐term elevation of glucocorticoids, would increase the probability of mice exhibiting subordinate behavior when exposed to new colonies because of reduced levels of aggression. However, this was not the case, either because CSDS was not as stressful for these animals, especially considering they did not show any signs of anhedonia or increased state anxiety, or because the animals used other strategies to develop a good social ranking. As we did not directly measure aggression during hierarchy formation, we cannot confirm how the aggression levels of CSDS‐exposed animals were compared to the stress‐naïve individuals. However, the relationship between glucocorticoid levels and social rank is complex and has been described as highly dependent on social context (Weger et al., 2018). We do not expect social isolation prior to CSDS to have had significant effects on glucocorticoids, as endocrine changes in isolated compared to group‐housed animals are very limited (Benton & Brain, 1981; Holson et al., 1991; Misslin et al., 1982).

To further determine the effects of stress on susceptible/resilient mice, we investigated whether diurnal activity was disrupted following CSDS exposure. Since a number of studies report that stress affects circadian rhythms and sleep‐wake cycle (Bunney et al., 2015) we predicted that stress exposure following CSDS would lead to disruptions in daily rhythms. Daily wheel‐running activity is a standard measure of internal rhythms where mice will typically exhibit low activity in the day and high activity at night. We did not observe any obvious effect of CSDS on total activity counts in daily wheel‐running activity since both susceptible and resilient mice exhibited similar rhythms to stress‐naïve mice.

While we report measures of sociability, anxiety, and locomotive behavior, it is nonetheless difficult to account for the complete array of side‐effects that CSDS may have on cognition and physiology, and how these in turn may affect social dominance. Though our initial study measured the effect of stress on winning ratios in completely new social groups, another valuable question would be to examine how stress would affect integration of an individual in an already established group of stress‐naïve conspecifics.

### 4.2 Dominant and subordinate mice are equally susceptible to the adverse effects of chronic social stress

The differences in social competitiveness were not related to the overall differences in stress susceptibility. However, subordinate, but not dominant, mice exhibited decreased average baseline social preference prior to CSDS. This may be indicative of social stressors experienced by these groups during hierarchy formation which coincides with recent suggestions of high levels of intrinsic stress in selectively bred socially submissive mice (Bairachnaya et al., 2019). Repeatedly defeated males showed classical stress responses such as decreased body weight gain, increased adrenals, and increased basal corticosterone levels (Haller et al., 1999). Like repeated defeat from aggressive residents in CSDS, low social status in social groups can also induce chronic stress in male rodents (Blanchard et al., 1995). Therefore, it is not surprising that subordinate males are already showing signs of chronic stress after 2 weeks of group‐housing. CSDS following 2 weeks of group housing likely added further stress, driving the subordinate population to even lower sociability which indicates increased social stress. However, both hierarchical groups exhibited susceptible phenotypes as evidenced by their comparably low social interaction scores.

Defeat involves the loss of social status which would be more pertinent for those males that enjoyed a higher social status in original colonies, than for males who already lost and attained only low dominance over other group members. A recent study reported that dominant males were indeed more susceptible to CSDS (Larrieu et al., 2017) while we found that dominant mice exhibited the greater change in sociability when we compared SI scores before and after CSDS relative to the subordinates. However, we did not find any evidence that the dominants were more stressed than the subordinates. In comparison, Larrieu et al. used 5 weeks of cohabitation prior to dominance testing and CSDS, while we opted for 2 weeks of cohabitation as it has been shown to be sufficient for hierarchy formation. Therefore, the inconsistency in stress susceptibility may arise because the effects of group‐housing are relatively mild at 2 weeks and/or because more profound effects are only observable after prolonged occupation of a certain rank. One hypothesis may be that the dominants suffer more severe stress when having to maintain their position throughout a longer period, while during the initial establishment of hierarchy the subordinates experience more stress. Our findings support this hypothesis since subordinate mice already exhibited a susceptible‐like sociability phenotype prior to introduction of any additional stressors as evidenced by the significantly lower pre‐CSDS SI ratio. Another recent study also reported subordinate mice having higher depressive‐like behavior, as well as hormonal and expression levels of genes associated with stress, after only 2 weeks of group‐housing (Horii et al., 2017). As baseline SI measurements were not reported by Larrieu et al. we cannot make a direct comparison with hierarchies maintained for a longer period.

It is possible that increased vulnerability to chronic stressors may be an effect of long‐term dominance, arising from the struggles to maintain the rank position, similar to how long‐term subordination in the visible‐burrow system induces a stress‐phenotype (Blanchard et al., 1995). Nonetheless, studies on hierarchy maintenance in mice showed that there was a large degree of variability between social groups in overall stability, time taken in establishing the hierarchy and in the degree of despotism of the alpha male (Williamson et al., 2016). As a result, we would expect rank‐related differences to arise over a variable timescale, making duration of group‐housing a significant contributor to the effects of social hierarchies on behavior and physiology. Moreover, another explanation for the differences between the studies may be due to the type and the strength of stressors used. Acute and chronic stressors can have very different effects on neurophysiological function where, for example, strong stressors lead to increased firing, but longer term weaker stressors induced decreased firing in the brain reward circuits (Chaudhury et al., 2013; Tye et al., 2013; Venzala et al., 2013). Thus, differences in the stress paradigms used in these studies likely induced different changes in neural circuits that will have different effects on behavioral processes such as motivation and aggression during hierarchy formation resulting in variations in observed responses (Zhou et al., 2017).

It was recently reported that dominant mice exhibited increased sleep fragmentation which were hypothesized to be due to the stressful effects of having to constantly maintain a dominant status (Karamihalev et al., 2019). Since sleep and circadian rhythms are intimately linked, we wondered whether dominant and subordinate mice would exhibit different daily rhythms following stress exposure. However, analysis of daily wheel‐running activity did not show any difference in diurnal total activity counts per hour in dominant and subordinate mice.

## 5 CONCLUSIONS

In summary, our results suggest that social status was not determined by prior exposure to chronic stress. Specifically, mice that are susceptible or resilient to chronic social stressors were as likely to be dominant or subordinate when exposed to new groups of animals that did not have any dominance experience for at least 1 week prior to the stress exposure. In contrast, the continuous stress of establishing and maintaining a hierarchy had differential effects on mice of distinct ranks. In newly established groups living together for at least 2 weeks, low‐status, subordinate mice exhibited lower preference to novel social targets, but, after the exposure to chronic social stress, on the sociability of low‐status animals was comparable to that of high‐status animals.

The clinical consequences of social stress are increasing as the number of people living in urban settings increases together with modern life–work demands. Thus, there is a need to expand our knowledge of stress‐related factors influencing social behaviors for the purposes of developing appropriate therapeutics. Animal models currently assume that shared housing implies greater phenotypic similarity between animals, but recent studies show how social dominance accounted for more variation in mice than cage identity (Varholick et al., 2019). Refinement of such basic assumptions in animal behavior research would be necessary to systematically study the behavioral and physiological consequence of social stress.

## CONFLICT OF INTEREST

None.

## AUTHOR CONTRIBUTIONS

Merima Šabanović conceived the study; designed the methodology; was involved in the investigation and formal analysis; and wrote, reviewed, and edited the original draft of the manuscript. He Liu designed the methodology, and investigated and supervised the study. Vongai Mlambo was involved in validation and investigation, and reviewed the original draft of the manuscript. Hala Aqel was involved in investigation. Dipesh Chaudhury was involved in conceptualization and funding acquisition, and reviewed and edited the original draft of the manuscript.

### Peer Review

The peer review history for this article is available at https://publons.com/publon/10.1002/brb3.1896.

## Supporting information

Fig S1

Fig S2

## Data Availability

The authors declare that the data supporting the findings of this study are available on request from the corresponding author (D.C.).
