# Supplementary figures and images for: What it takes to be at the top: The interrelationship between chronic social stress and social dominance
Source: Brain Behav. 2020 Oct 17;10(12):e01896. doi: 10.1002/brb3.1896 (PMC7749537; doi:10.1002/brb3.1896)

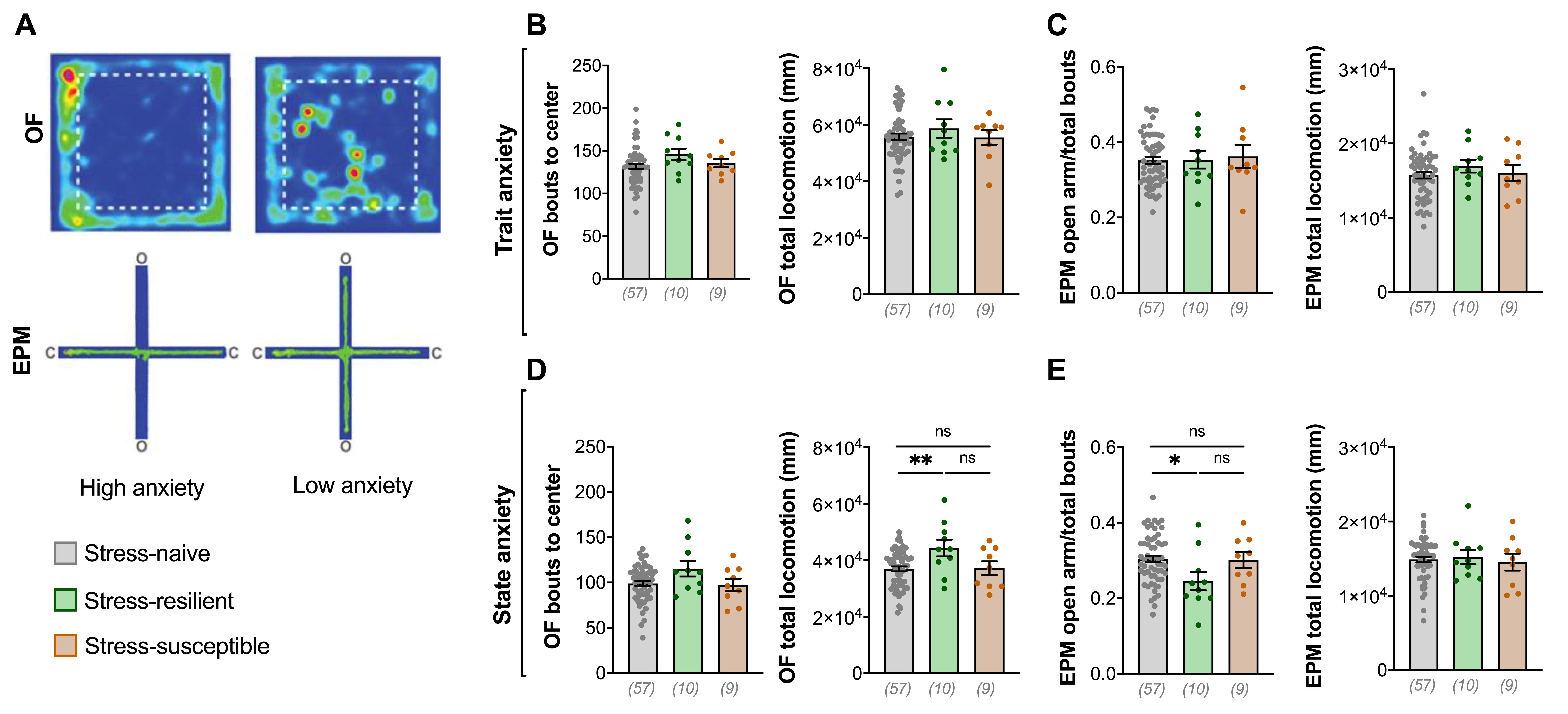

Supplement: Supplementary file 1 — Fig S1 [file BRB3-10-e01896-s001.jpg]

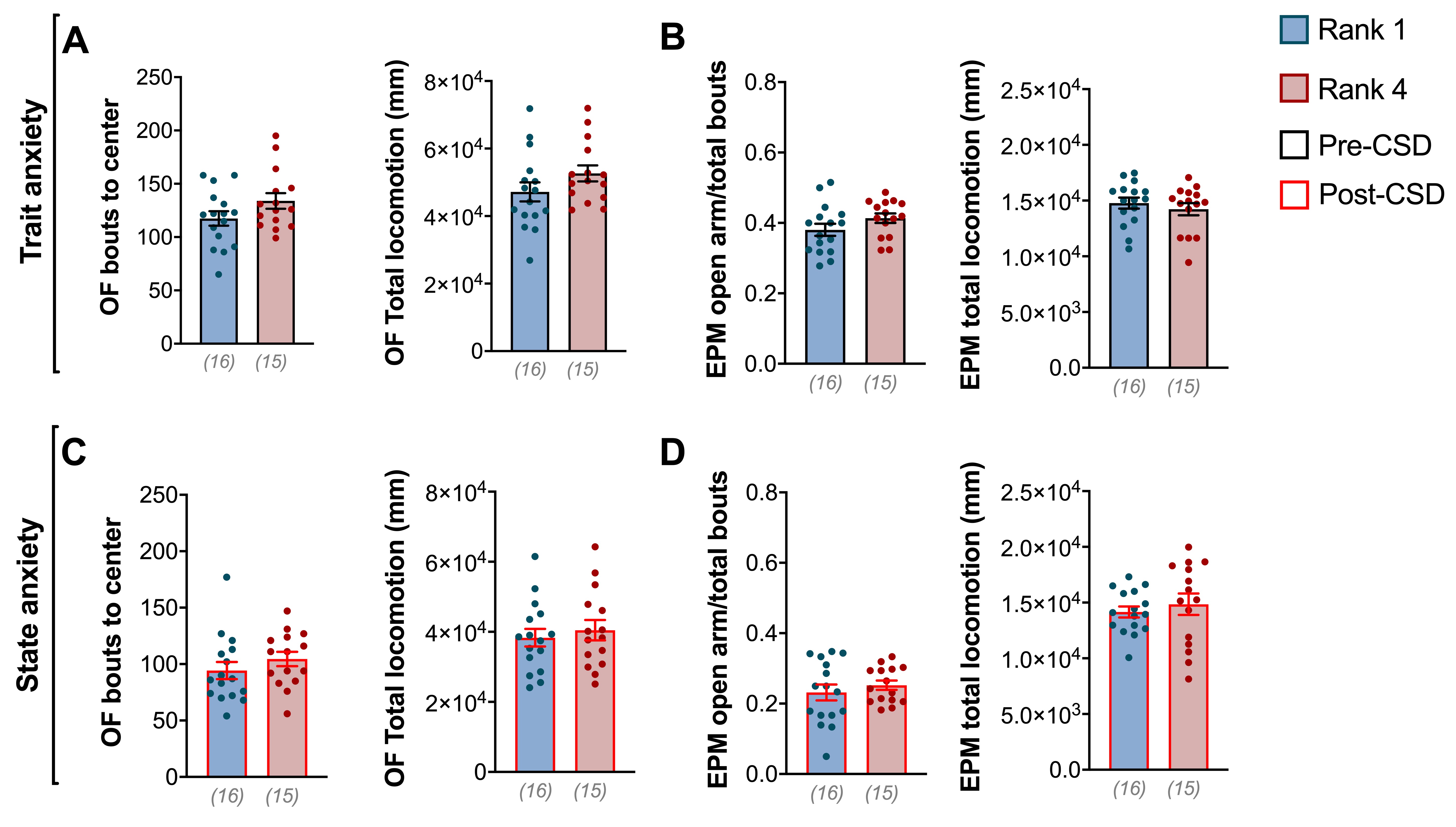

Supplement: Supplementary file 2 — Fig S2 [file BRB3-10-e01896-s002.jpg]
